# Occupational exposure to carcinogenic substances in night versus day shift jobs

**DOI:** 10.5271/sjweh.4307

**Published:** 2026-07-01

**Authors:** Jaclyn Parks, Umaimah Zanif, Parveen Bhatti

**Affiliations:** 1 Population Health Sciences, BC Cancer Research Institute, Vancouver, BC, Canada.; 2 School of Population and Public Health, University of British Columbia, Vancouver, BC, Canada.

**Keywords:** carcinogen, co-exposure, hazard, night shift, night work, shift work, shift worker

## Abstract

**Objectives:**

This study aimed to determine whether night shift work was associated with exposure to carcinogenic hazards, independent of occupation and industry.

**Methods:**

We conducted a cross-sectional analysis of 16 250 job records reported by 7023 participants. Participants provided lifetime job histories, including shift schedules and exposures to hazardous agents. Carcinogenic agents included established carcinogens classified by the International Agency for Research on Cancer, as well as broader exposure categories that may potentially contain carcinogenic constituents. Jobs involving ≥1 and ≥7 night shifts/month were compared with day-shift jobs. Occupations and industries were coded using National Occupational Classification (2016) and North American Industry Classification System (2017) codes. Generalized linear mixed models with random intercepts for participant, occupation code (2-digit level), and industry code (2-digit level) were used to estimate odds ratios (OR) for exposures associated with night- versus day-shift work, adjusting for biological sex and job start year.

**Results:**

Night shifts were associated with greater odds of any hazardous exposure [≥1 night shift/month: OR 3.1, 95% confidence interval (CI) 2.6–3.8; ≥7 night shifts/month: OR 3.1, 95% CI 2.5–3.8]. Significant associations were observed for multiple agents, including asbestos, degreasing agents, diesel engine exhaust, gasoline engine exhaust, ionizing radiation, mineral/cutting/lubricating oil, paints/stains/varnish, welding fume, and wood dust.

**Conclusions:**

Night-shift work was associated with an increased likelihood of self-reported exposure to multiple potentially carcinogenic agents, independent of broad occupational and industry groupings. Future research with more granular task-level information and quantitative exposure assessment will be important for clarifying the mechanisms underlying these differences.

Night shift work has been linked to increased risks of cancer (1). However, the specific aspects of night shifts that drive their carcinogenic impact remain poorly understood, thereby limiting the development of effective prevention strategies. The underlying causes likely involve multiple interacting factors such as circadian disruption, light at night, and sleep deprivation (2). A potential contributor to increased cancer risks among night shift workers that has received comparatively little attention involves differential exposure to hazardous substances.

Few studies have compared the prevalence of exposure to hazards between night and day shift workers, but most have found evidence that night shift workers are more likely to encounter hazardous substances in the workplace (3–7). What remains uncertain is whether night shift schedules themselves contribute to elevated exposure, independent of job type. In other words, it is unclear whether working at night inherently increases the likelihood of encountering hazardous substances, or if the observed differences simply reflect the kinds of jobs that are more likely to entail night shift work. While some studies have attempted to adjust for differences in job type (3, 4), the occupational groups used in the analyses were broad and may not have captured the substantial variation in exposure that exists across specific occupations and industries. Analyses that account for occupation and industry at a more granular level may help disentangle the independent contribution of night shift schedules to carcinogenic exposures.

To address this gap in knowledge, we leveraged detailed lifetime occupational histories from a subset of participants in the BC Generations Project (BCGP) (8). These data allowed us to examine whether night shift work is associated with a higher likelihood of occupational exposure to hazardous substances, while accounting for differences in occupation and industry. By characterizing how night and day shift work differ in terms of occupational exposures, this study contributes to a more complete understanding of the potential pathways through which night shift work may influence cancer risk.

## Methods

The University of British Columbia/BC Cancer Research Ethics Board approved this study (H25-03169).

### Study population

The BCGP is a population-based cohort consisting of 29 736 adults from British Columbia aged 35–69 years at enrollment in 2009–2016 (8). At recruitment, after giving informed consent, participants completed an extensive questionnaire that collected information on demographics, lifestyle factors, and health history.

### Occupational histories

In 2018, 18 364 BCGP participants completed an online occupational history questionnaire. For every job they held for ≥3 months, participants were asked to report multiple details, including: start and end year, type of industry, job title, most common shift schedule (regular morning, regular day, regular evening, regular night, rotating, split, irregular, other) and, if applicable, number of night shifts worked per month. In addition, for each job held, participants reported whether they had ever been exposed to a list of potentially hazardous agents. Potentially carcinogenic agents included: asbestos, benzidine, benzene, cadmium salts, chromium salts, coal tar/soot/pitch/creosote, degreasing agents, diesel engine exhaust, dyestuffs, gasoline engine exhaust, glues, herbicides/insecticides/fungicides, ionizing radiation, mineral/cutting/lubricating oils, paints/stains/varnishes, pressure-treated wood, vinyl chloride, welding fumes, and wood dust. Several of these agents were selected because the International Agency for Research on Cancer has classified them as carcinogenic to humans (Group 1) (9). Others were selected because they are broader classes of exposures that may contain carcinogenic constituents.

A total of 101 548 records were completed within these questionnaires. For this study, all jobs involving ≥1 night/month of night shift work were selected for inclusion (N=11 250). In addition, a random sample of 5000 day shift job records was included for comparison. Relevant items from the occupational history questionnaire, including shift schedule and exposure questions, are provided in the supplementary material (www.sjweh.fi/article/4307).

### Occupation and industry coding

Between 2022 and 2025, the 16 250 job records included in this study were coded for occupation and industry. A total of eight trained coders carried out the coding during this period, with three to four coders working concurrently at any given time. Job codes (≤4 digits) were assigned based on the National Occupational Classification (NOC) 2016 version 1.1 and industry codes (≤6 digits) were assigned based on the North American Industry Classification System (NAICS) Canada 2017 version 3.0. Coders were instructed to flag any records they were uncertain about and add them to a shared database for review. Coders met at least monthly to discuss these uncertain records and to resolve any discrepant codes from the ~10% of records that were blindly coded by at least two coders to assess coding consistency. Final coding decisions were made by consensus during these meetings.

To support stable estimation at the level used in analysis, occupation and industry codes were aggregated into 2-digit groupings. Within these 2-digit categories, groups with small numbers of observations were further merged with conceptually similar categories for analysis. The ‘management of companies and enterprises’ industry group was merged with the ‘finance and insurance’ industry group. The ‘assemblers in manufacturing’ and ‘installers, repairers and servicers and material handlers’, and ‘processing, manufacturing and utilities supervisors and central control operators’ occupation groups were merged with the ‘labourers in processing, manufacturing and utilities’ group. The ‘finance, insurance and related business administration’ occupation group was merged with the ‘professional occupations in business and finance’ occupation group, and the ‘occupations in front-line public protection services’ group was merged with the ‘care providers and educational, legal and public protection support’ group. Finally, the ‘professional occupations in business and finance’ group was merged with the ‘administration and financial supervisors’ occupation group.

### Statistical analysis

For the duplicate coded records, Cohen’s kappa coefficients were calculated for each coder at the two-digit level of the NOC and NAICS (only the first two digits were considered for this study since many job/industry categories based on >2-digits were sparsely populated when stratifying by shift status), comparing the coder’s assigned code against the consensus code. To provide a summary measure of coding consistency, the kappa values were averaged across coders.

We used generalized linear mixed models to evaluate associations between night shift status and workplace hazardous exposures. Because participants could report multiple jobs over their working lifetimes, a random intercept for participant was included to account for correlation between jobs held by the same individual. All models were additionally adjusted for biological sex and year of job start (categorical: 1945–1972, 1973–1980, 1981–1991, or 1992–2018) as fixed effects. To address potential confounding by occupation and industry, we included random intercepts for 2-digit NOC and NAICS codes, respectively.

The primary fixed effect of interest was night shift status (≥1 night shift per month versus day shift). Models were fit separately for any hazardous exposure and individual classes of hazardous substances (binary outcomes, logit link), and the total number of distinct hazardous exposures reported for a job (count outcome, negative binomial link to account for overdispersion). We repeated all analyses excluding those night/rotating shift jobs that reported less than the median number of night shifts/month. Odds ratios (OR) and 95% confidence intervals (CI) were reported for the binary outcomes, while exposure count ratios (ECR) and 95% CI were reported for the count outcome. All analyses were conducted in R (version 4.5.0) using the lme4 and glmmTMB packages.

## Results

The mean Cohen’s kappa at the 2-digit level was 0.89 [standard deviation (SD) 0.03] for NOC codes and 0.92 (SD 0.02) for NAICS codes, indicating a high degree of reliability in the occupation and industry coding process. The 16 250 job records included in this analysis represented 7023 participants, with an average of 2.3 (SD 2.1) records per person. Most participants were female (63%), and non-Hispanic white (86%). At the time of occupational history questionnaire completion, participants were an average of 63.0 (SD 8.4) years of age.

Among jobs involving ≥1 night shift/month, the mean and median number of night shifts /month were 8.4 (SD 6.3) and 7, respectively. A greater proportion of day shift jobs had more recent start years (30.8% began between 1992 and 2018) compared with jobs involving ≥1 night shift/month (20.6%) or ≥7 night shifts/month (16.4%) (table 1). Job duration did not differ meaningfully across shift categories. A total of 20 industry groups were identified. Among day shift jobs, the most frequently assigned industry was ‘professional, scientific & technical services’. In contrast, ‘health care & social assistance’ was the most common industry for jobs involving ≥1 night shift/month (36.9%) and ≥7 night shifts/month (39.6%). Substantial differences in industry distribution were observed between day and night shift jobs. For example, 14.8% of day shift jobs were classified as ‘educational services,’ compared with only 2.2 and 1.3% of jobs involving ≥1 or ≥7 night shifts/month, respectively.

**Table 1:** Job level characteristics stratified by shift status.

Characteristic		Day shift		Night shift		
				≥ 1 shift/month		≥7 shifts/month
		N (%)		N (%)		N (%)
**Start year**						
	1945–1972	1094 (21.9)		3015 (26.8)		1934 (30.6)
	1973–1980	1057 (21.1)		2998 (26.6)		1793 (28.4)
	1981–1991	1308 (26.2)		2920 (26.0)		1551 (24.6)
	1992–2018	1541 (30.8)		2317 (20.6)		1037 (16.4)
**Duration (years)**						
	≤2	1751 (35.0)		3647 (32.4)		2254 (35.7)
	>2–≤4	1169 (23.4)		2832 (25.2)		1556 (24.6)
	>4–≤8	888 (17.8)		1917 (17.0)		991 (15.7)
	>8	1192 (23.8)		2854 (25.4)		1514 (24.0)
**Industry**						
	Accommodation & food services	149 (3.0)		967 (8.6)		532 (8.4)
	Administration & support, waste management remediation services	168 (3.4)		227 (2.0)		116 (1.8)
	Agriculture, forestry, fishing and hunting	68 (1.4)		144 (1.3)		82 (1.3)
	Arts, entertainment & recreation	89 (1.8)		226 (2.0)		98 (1.6)
	Construction	121 (2.4)		180 (1.6)		71 (1.1)
	Educational services	741 (14.8)		244 (2.2)		82 (1.3)
	Finance & insurance	331 (6.6)		90 (0.8)		46 (0.7)
	Health care & social assistance	678 (13.6)		4150 (36.9)		2505 (39.6)
	Information & cultural industries	220 (4.4)		430 (3.8)		162 (2.6)
	Mgmt. of companies & enterprises	21 (0.4)		5 (0.04)		2 (0.0)
	Manufacturing	345 (6.9)		1210 (10.8)		884 (14.0)
	Mining, quarrying, & oil and gas extraction	56 (1.1)		245 (2.2)		209 (3.3)
	Other services	191 (3.8)		183 (1.6)		76 (1.2)
	Professional, scientific & technical services	755 (15.1)		439 (3.9)		142 (2.2)
	Public administration	420 (8.4)		1014 (9.0)		556 (8.8)
	Real estate & rental and leasing	55 (1.1)		53 (0.5)		28 (0.4)
	Retail trade	317 (6.3)		655 (5.8)		236 (3.7)
	Transportation & warehousing	122 (2.4)		647 (5.8)		418 (6.6)
	Utilities	30 (0.6)		54 (0.5)		27 (0.4)
	Wholesale trade	123 (2.5)		87 (0.8)		43 (0.7)
**Occupation**						
	Administration & financial supervisors	521 (10.4)		172 (1.5)		77 (1.2)
	Assemblers in manufacturing	13 (0.3)		44 (0.4)		33 (0.5)
	Assisting occupations supporting health services	60 (1.2)		317 (2.8)		182 (2.9)
	Care providers & educational, legal & public protection support	64 (1.3)		209 (1.9)		108 (1.7)
	Distribution, tracking and scheduling coordination	59 (1.2)		187 (1.7)		140 (2.2)
	Finance, insurance & related business administration	36 (0.7)		14 (0.1)		4 (0.1)
	Harvesting, landscaping & natural resources laborers	43 (0.9)		82 (0.7)		59 (0.9)
	Industrial, electrical & construction trades	64 (1.3)		227 (2.0)		113 (1.8)
	Installers, repairers & servicers & material handlers	12 (0.2)		112 (1.0)		77 (1.2)
	Labourers in processing, manufacturing & utilities	31 (0.6)		452 (4.0)		346 (5.5)
	Maintenance & equipment operation trades	26 (0.5)		226 (2.0)		157 (2.5)
	Middle management in retail, wholesale trade & customer services	101 (2.0)		256 (2.3)		97 (1.5)
	Middle management in trades, transporation, production & utilities	60 (1.2)		94 (0.8)		30 (0.5)
	Occupation in front-line public protection services	7 (0.1)		393 (3.5)		243 (3.8)
	Office support	536 (10.7)		327 (2.9)		164 (2.6)
	Paraprofessional in legal, social, community & government services	116 (2.3)		212 (1.9)		80 (1.3)
	Processing & manufacturing machine operators & related production	33 (0.7)		227 (2.0)		186 (2.9)
	Processing, manufacturing & utilities supervisors & central control operators	9 (0.2)		154 (1.4)		111 (1.8)
	Professional occupation in art & culture	127 (2.5)		150 (1.3)		42 (0.7)
	Professional occupation in business & finance	205 (4.1)		49 (0.4)		14 (0.2)
	Professional occupation in education services	506 (10.1)		143 (1.3)		46 (0.7)
	Professional occupation in health (not nursing)	107 (2.1)		464 (4.1)		232 (3.7)
	Professional occupation in law & social, community and government services	262 (5.2)		135 (1.2)		45 (0.7)
	Professional occupation in natural & applied sciences	313 (6.3)		252 (2.2)		69 (1.1)
	Professional occupation in nursing	86 (1.7)		2452 (21.8)		1693 (26.8)
	Retail sales supervisor & specialist sales	50 (1.0)		57 (0.5)		35 (0.6)
	Sales representatives & salespersons in wholesale & retail trade	132 (2.6)		151 (1.3)		49 (0.8)
	Sales support	118 (2.4)		370 (3.3)		158 (2.5)
	Senior management	104 (2.1)		83 (0.7)		20 (0.3)
	Service representative & other customer & personal services	179 (3.6)		716 (6.4)		410 (6.5)
	Service supervisors & specialized service	50 (1.0)		212 (1.9)		110 (1.7)
	Service support & other service occupation	73 (1.5)		303 (2.7)		178 (2.8)
	Specialized middle management	420 (8.4)		340 (3.0)		113 (1.8)
	Supervisors and technical occupation in natural resources, agriculture & related production	8 (0.2)		69 (0.6)		58 (0.9)
	Technical occupation in art, culture, recreation & sport	122 (2.4)		193 (1.7)		81 (1.3)
	Technical occupation in health	105 (2.1)		501 (4.5)		228 (3.6)
	Technical occupation related to natural & applied sciences	157 (3.1)		494 (4.4)		242 (3.8)
	Trades helpers, construction labourers & related occupation	36 (0.7)		69 (0.6)		45 (0.7)
	Transport, heavy equipment & related maintenance	23 (0.5)		242 (2.2)		173 (2.7)
	Workers in natural resources, agriculture & related production	26 (0.5)		100 (0.9)		67 (1.1)

A total of 40 occupation groups were identified (table 1). The most common occupation among day shift jobs was ‘office support,’ whereas ‘professional occupations in nursing’ were most common among jobs involving ≥1 night shift/month (21.8%) and/or ≥7 night shifts/month (26.8%). Marked differences in occupational distributions were evident across shift categories. For instance, 10.4% of day shift jobs were classified as ‘administrative and financial supervisors’, compared with only 2.2 and 1.3% of jobs involving ≥ 1 or ≥7 night shifts/month, respectively.

Exposure to at least one potentially carcinogenic agent was reported for 31.9% of jobs involving ≥1 night shift/month and 35.7% of jobs involving ≥7 night shifts/month, compared with 12.1% of day shift jobs (table 2). The mean total number of hazards to which exposure was reported was 0.99 (SD 2.1) and 1.1 (SD 2.2) for jobs involving ≥1 night shift/month and jobs involving ≥7 night shifts/month, respectively, compared with 0.30 (SD 1.1) for day shift jobs. For most individual agents, reported exposure was more common among night shift jobs than day shift jobs and was slightly more frequent among jobs involving ≥7 night shifts/month than those involving ≥1 night shift/month. For example, asbestos exposure was reported for 1.5% of day shift jobs, compared with 7.2% of jobs involving ≥1 night shift/month and 8.3% of jobs involving ≥7 night shifts/month. Although exposures to benzidine, cadmium salts, chromium salts, and dyestuffs were more frequently reported among night shift jobs, these agents were excluded from individual statistical analyses due to the small number of exposed day shift jobs (eg, N=4 day shift jobs reported benzidine exposure).

**Table 2:** Potentially carcinogenic exposures by shift status.

Exposure		Day shift		Night shift		
				≥1 shift/month		≥7 shifts/month
		N (%)		N (%)		N (%)
Any						
	Never	4395 (87.9)		7656 (68.0)		4062 (64.3)
	Ever	605 (12.1)		3594 (31.9)		2253 (35.7)
Asbestos						
	Never	4923 (98.5)		10 437 (92.8)		5793 (91.7)
	Ever	77 (1.5)		813 (7.2)		522 (8.3)
Benzidine						
	Never	4996 (99.9)		11 150 (99.1)		6257 (99.1)
	Ever	4 (0.1)		100 (0.9)		58 (0.9)
Benzene						
	Never	4933 (98.7)		10 983 (97.6)		6160 (97.5)
	Ever	67 (1.3)		267 (2.4)		155 (2.5)
Cadmium salts						
	Never	4988 (99.8)		11 191 (99.5)		6280 (99.4)
	Ever	12 (0.2)		59 (0.5)		35 (0.6)
Chromium salts						
	Never	4982 (99.6)		11 186 (99.4)		6278 (99.4)
	Ever	18 (0.4)		64 (0.57)		37 (0.6)
Coal tar, soot, pitch, asphalt, creosote						
	Never	4937 (98.7)		10 618 (94.4)		5932 (93.9)
	Ever	63 (1.3)		632 (5.6)		383 (6.1)
Degreasing agents						
	Never	4934 (98.7)		10 558 (93.8)		5867 (92.9)
	Ever	66 (1.3)		692 (6.2)		448 (7.1)
Diesel engine exhaust						
	Never	4870 (97.4)		9755 (86.7)		5351 (84.7)
	Ever	130 (2.6)		1495 (13.2)		964 (15.3)
Dyestuffs						
	Never	4978 (99.6)		11 118 (98.8)		6249 (99.0)
	Ever	22 (0.4)		132 (1.2)		66 (1.0)
Gasoline engine exhaust						
	Never	4836 (96.7)		9907 (88.1)		5504 (87.2)
	Ever	164 (3.3)		1343 (11.9)		811 (12.8)
Glue						
	Never	4874 (97.5)		10 723 (95.3)		6032 (95.5)
	Ever	126 (2.5)		527 (4.7)		283 (4.5)
Herbicides, insecticides, fungicides						
	Never	4929 (98.6)		10 957 (97.4)		6160 (97.5)
	Ever	71 (1.4)		293 (2.6)		155 (2.5)
Ionizing radiation						
	Never	4913 (98.3)		10 402 (92.5)		5801 (91.9)
	Ever	87 (1.7)		848 (7.5)		514 (8.1)
Mineral, cutting, lubricating oil						
	Never	4897 (97.9)		10 291 (91.5)		5699 (90.2)
	Ever	103 (2.1)		959 (8.5)		616 (9.8)
Paints, stains, varnish						
	Never	4812 (96.2)		10 417 (92.6)		5835 (92.4)
	Ever	188 (3.8)		833 (7.4)		480 (7.6)
Pressure-treated wood						
	Never	4938 (98.8)		10 960 (97.4)		6162 (97.6)
	Ever	62 (1.2)		290 (2.6)		153 (2.4)
Vinyl chloride						
	Never	4991 (99.8)		11 131 (98.9)		6252 (99.0)
	Ever	9 (0.2)		119 (1.1)		63 (1.0)
Welding						
	Never	4907 (98.1)		10 532 (93.6)		5844 (92.5)
	Ever	93 (1.9)		718 (6.4)		471 (7.5)
Wood dust						
	Never	4843 (96.9)		10 331 (91.8)		5722 (90.6)
	Ever	157 (3.1)		919 (8.2)		593 (9.4)

In models adjusted for biological sex and job start year, jobs involving ≥1 night shift/month were associated with a statistically significant 4.1-fold increased odds of exposure to any potentially carcinogenic agent compared to day shift jobs (95% CI 3.3–5.1; table 3). Jobs involving ≥7 night shifts/month had a statistically significant 5.2-fold increased odds (95% CI 4.1–7.1). After further adjustment for industry and occupation groups, OR were attenuated but remained significantly elevated for both jobs involving ≥1 night shift/month (OR 3.1, 95% CI 2.6–3.8) and ≥7 night shifts/month (OR 3.1, 95% CI 2.5–3.8), relative to day shift jobs.

**Table 3:** Odds of exposure to potentially carcinogenic agents for jobs involving ≥1 and ≥7 night shifts/month as compared to day shift jobs. [OR=odds ratio; CI=confidence interval.]

Exposure		≥1 night shift/month			≥7 night shifts/month	
		OR (95% CI) ^a^	OR (95% CI) ^b^		OR (95% CI) ^a^	OR (95% CI) ^b^
Any						
	Never	Ref.	Ref.		Ref.	Ref.
	Ever	4.1 (3.3–5.1)	3.1 (2.6–3.8)		5.2 (4.1–7.1)	3.1 (2.5–3.8)
Asbestos						
	Never	Ref.	Ref.		Ref.	Ref.
	Ever	4.2 (1.8–9.8)	6.2 (2.4–16.0)		4.4 (1.7–11.4)	3.8 (1.3–11.1)
Benzene						
	Never	Ref.	Ref.		Ref.	Ref.
	Ever	1.1 (0.4–2.7)	0.9 (0.3–2.6)		1.0 (0.3–3.0)	1.7 (0.4–6.8)
Coal tar, soot, pitch, asphalt, creosote						
	Never	Ref.	Ref.		Ref.	Ref.
	Ever	2.4 (1.0–5.5)	2.2 (0.8–6.2)		2.0 (1.4–2.9)	2.1 (0.6–7.4)
Degreasing agents						
	Never	Ref.	Ref.		Ref.	Ref.
	Ever	2.7 (1.2–5.8)	4.2 (2.8–6.4)		3.2 (1.2–8.1)	4.2 (1.5–12.2)
Diesel engine exhaust						
	Never	Ref.	Ref.		Ref.	Ref.
	Ever	6.2 (3.4–11.2)	6.3 (4.8–8.3)		3.7 (1.8–7.2)	7.9 (5.8–10.7)
Gasoline engine exhaust						
	Never	Ref.	Ref.		Ref.	Ref.
	Ever	3.9 (2.3–6.8)	3.8 (2.9–4.9)		3.8 (1.8–7.6)	4.2 (3.2–5.6)
Glue						
	Never	Ref.	Ref.		Ref.	Ref.
	Ever	1.1 (0.6–2.0)	1.5 (0.7–3.0)		0.9 (0.4–1.9)	0.7 (0.3–1.8)
Herbicides, insecticides, fungicides						
	Never	Ref.	Ref.		Ref.	Ref.
	Ever	1.1 (0.5–2.3)	2.5 (0.9–6.6)		1.0 (0.4–2.7)	1.2 (0.4–4.0)
Ionizing radiation						
	Never	Ref.	Ref.		Ref.	Ref.
	Ever	16.2 (6.4–40.6)	9.3 (3.5–24.9)		14.5 (3.5–59.9)	6.7 (1.5–29.5)
Mineral, cutting, lubricating oil						
	Never	Ref.	Ref.		Ref.	Ref.
	Ever	3.0 (1.5–5.8)	5.5 (2.5–12.1)		3.3 (1.5–7.1)	2.7 (1.1–6.6)
Paints, stains, varnish						
	Never	Ref.	Ref.		Ref.	Ref.
	Ever	1.0 (0.6–1.7)	1.8 (1.4–2.2)		0.8 (0.4–1.6)	1.6 (1.2–2.1)
Pressure-treated wood						
	Never	Ref.	Ref.		Ref.	Ref.
	Ever	0.9 (0.4–2.1)	1.1 (0.4–3.4)		0.8 (0.3–2.3)	0.5 (0.2–1.9)
Welding fume						
	Never	Ref.	Ref.		Ref.	Ref.
	Ever	2.0 (0.9–4.3)	3.0 (2.1–4.4)		2.0 (0.8–5.0)	3.8 (2.5–5.6)
Wood dust						
	Never	Ref.	Ref.		Ref.	Ref.
	Ever	1.5 (0.9–2.5)	1.8 (1.4–2.4)		1.1 (0.6–2.0)	1.8 (1.3–2.5)

ª Adjusted for participant, biological sex, and job start year. ^b^ Adjusted for participant, biological sex, job start year, industry group, and occupation group.

No statistically significant evidence of associations between night shift work and exposure to benzene, glues, herbicides/insecticides/fungicides, or pressure-treated wood was observed (table 3). The strongest associations were observed for ionizing radiation. In models adjusted for biological sex and job start year, jobs involving ≥1 night shift/month had a 16.2-fold greater odds of exposure to ionizing radiation compared with day shift jobs (95% CI 6.4–40.6), while jobs involving ≥7 night shifts/month had a 14.5-fold greater odds (95% CI 3.5–59.9). After additional adjustment for industry and occupation groups, odds of ionizing radiation exposure were attenuated though remained statistically significant for jobs involving ≥1 night shift/month (OR 9.3, 95% CI 3.5–24.9) and ≥7 night shifts/month (OR 6.7, 95% CI 1.5–29.5), compared with day shift jobs.

Several additional agents demonstrated strong and consistent positive associations with night shift work. Jobs involving ≥1 night shift/month had elevated odds of exposure to asbestos in models adjusted for sex and start year (OR 4.2, 95% CI 1.8–9.8) and after further adjustment for industry and occupation (OR 4.4, 95% CI 1.7–11.4). Jobs involving ≥7 night shifts/month also had elevated odds of exposure to asbestos in both minimally and fully adjusted models (OR 6.2 and 3.8, 95% CI 2.4–16.0 and 1.3–11.1, respectively). Exposure to degreasing agents was significantly associated with ≥1 night shift/month (OR 2.7, 95% CI 1.2–5.8) and ≥7 night shifts/month (OR 3.2, 95% CI 1.2–8.1) in models adjusted for biological sex and start year, with somewhat stronger associations after additional adjustment for industry and occupation (OR 4.2 and 4.2, 95% CI 2.8–6.4 and 1.5–12.2, respectively).

Exposure to diesel engine exhaust was strongly associated with night shift work (≥1 night shift/month: OR 6.2, 95% CI 3.4–11.2; ≥7 night shifts/month: OR 3.7, 95% CI 1.8–7.2), with associations remaining statistically significant after additional adjustment for industry and occupation (OR 6.3 and 7.9, 95% CIs 4.8–8.3 and 5.8–10.7, respectively). Odds of exposure to gasoline engine exhaust were also elevated among night shift jobs in both minimally adjusted (≥1 night shift/month: OR 3.9, 95% CI 2.3–6.8; ≥7 night shifts/month: OR 3.8, 95% CI 1.8–7.6) and fully adjusted models (OR 3.8 and 4.2, 95% CI 2.9–4.9 and 3.2–5.6, respectively).

Mineral/cutting/lubricating oils also showed consistent and statistically significant associations with night shift work. Increased odds of exposure were observed for jobs involving ≥1 night shift/month (OR 3.0, 95% CI 1.5–5.8) and ≥7 night shifts/month (OR 3.3, 95% CI 1.5–7.1) in minimally adjusted models and fully adjusted models (OR 5.5 and 2.7, 95% CI 2.5–12.1 and 1.1–6.6, respectively).

Exposure to coal tar/soot/pitch/asphalt/creosote was significantly associated with ≥1 night shift per month and ≥7 night shifts per month in models adjusted for sex and start year (OR 2.4, 95% CI 1.0–5.5 and OR 2.0, 95% CI 1.4–2.9). After additional adjustment for industry and occupation, the associations were no longer statistically significant (OR 2.2, 95% CI 0.8–6.2 and OR 2.1, 95% CI 0.6–7.4). Elevated odds of exposure to welding fumes (OR 3.0, 95% CI 2.1–4.4 for ≥1 night shift/month; OR 3.8, 95% CI 2.5–5.6 for ≥7 night shifts/month), wood dust (OR 1.8, 95% CI 1.4–2.4 for ≥1 night shift/month; OR 1.8, 95% CI 1.3–2.5 for ≥7 night shifts/month), and paints/stains/varnishes (OR 1.8, 95% CI 1.4–2.2 for ≥1 night shift/month; OR 1.6, 95% CI 1.2–2.1 for ≥7 night shifts/month), were observed only after adjustment for industry and occupation.

In models adjusted for biological sex and job start year, jobs involving ≥1 night shift/month were associated with a statistically significant 2.1-fold increased number of potentially carcinogenic exposures as compared to day shift jobs (95% CI 1.9–2.4; table 4). Jobs involving ≥7 night shifts/month were associated with a statistically significant 2.2-fold increased number of potentially carcinogenic exposures (95% CI 1.9–2.6). After further adjustment for industry and occupation groups, the ECR remained significantly elevated for both jobs involving ≥1 night shift/month (OR 2.2, 95% CI 1.9–2.5) and ≥7 night shifts/month (OR 2.0, 95% CI 1.8–2.4), relative to day shift jobs.

**Table 4:** Number of potentially carcinogenic exposures reported for jobs involving ≥1 and ≥7 night shifts/month as compared to day shift jobs. [ECR=exposure count ratio; CI=confidence interval.]

≥1 night shift/month			≥7 night shifts/month	
ECR (95% CI) ^a^	ECR (95% CI) ^b^		ECR (95% CI) ^a^	ECR (95% CI) ^b^
2.1 (1.9–2.4)	2.2 (1.9–2.5)		2.2 (1.9–2.6)	2.0 (1.8–2.4)

^a^ Adjusted for participant, biological sex, and job start year. ^b^ Adjusted for participant, biological sex, job start year, industry group, and occupation group.

## Discussion

Our analysis shows that jobs involving night shift work are associated with a greater likelihood of self-reported exposure to multiple potentially carcinogenic agents compared with day shift jobs. In fully adjusted models accounting for occupation and industry, jobs involving ≥1 night shift per month or ≥7 nights shift per month were significantly associated with exposure to: asbestos, degreasing agents, diesel engine exhaust, gasoline engine exhaust, ionizing radiation, mineral/cutting/lubricating oils, paints/stains/varnishes, welding fume, and wood dust. There was no compelling evidence that odds of exposure increased with more frequent night shifts when adjusting for industry and occupation groups. These findings suggest that jobs involving night shift work, independent of occupation or industry at a broad level, are associated with an increased likelihood of encountering specific potentially hazardous agents.

Our findings are generally similar with previous studies which have consistently shown that shift workers are more likely to experience multiple occupational hazards, although methods for accounting for occupational differences varied across studies. Jay et al (6) (N=3003) examined non-standard work hours and exposure to workplace hazards in a national survey of New Zealand workers but did not adjust for occupation or industry in multivariable models, raising the possibility that observed differences between standard and non-standard workers may have partially reflected occupational composition. Pepłońska et al (10) surveyed occupational hygienists across 44 industrial plants to characterize potential co-exposures associated with night shift work but did not conduct statistical analyses at the worker or job level; exposures were described at the enterprise level rather than linked to individual workers. Barber et al (7) (N=74 714) evaluated shift-related co-exposures using the French SUMER national occupational health surveys (2010 and 2017), but the findings were descriptive and did not include formal statistical testing of differences between shift groups or adjustment for occupation or industry. In the study by Miguet et al (4) (N=119 413), UK Biobank participants were grouped into nine 1-digit major occupational categories according to the UK Standard Occupational Classification 2000 (SOC2000). The study by El-Zaemey & Carey (3) (N=5425), which used data from a telephone survey study of Australian workers, similarly adjusted for ten major occupational groups. While these approaches captured broad occupational strata, adjustment at these levels may not have fully accounted for the heterogeneity in exposures within major job groups.

An increased likelihood of exposure to carcinogenic agents among jobs involving night shift work may reflect the organization of work within occupations and industries. Within the same industry and occupation categories, tasks involving maintenance, cleaning, equipment servicing, production processes, or diagnostic procedures that use hazardous agents may be disproportionately assigned to overnight hours. If such tasks are systematically concentrated during night shifts, workers on these schedules may experience greater opportunities for contact with hazardous substances independent of their overall job classification.

There is evidence that night shift workers may be more susceptible to the carcinogenic effects of occupational hazards. For example, night shift schedules have been linked to decreased efficiency in repairing oxidative DNA damage, a key pathway through which many chemical exposures exert mutagenic effects (11, 12). If night shift workers are both more likely to encounter hazardous agents and have diminished capacity to repair the resulting damage, their cumulative cancer risk may be elevated compared with that of day shift workers performing the same tasks. These findings could have important policy implications, highlighting the potential need for targeted exposure prevention strategies, enhanced occupational monitoring, and additional protective measures for night shift workers.

Strengths of this study include the use of a large, population-based cohort with detailed lifetime occupational histories, high reliability of occupation and industry coding, and the application of mixed models to account for within-person correlations and clustering by job type. Our study complements and extends the existing literature by examining exposures at the individual job level and incorporating more detailed adjustment for both occupation and industry using 2-digit classification codes. By analyzing individual job records rather than only participant-level classifications, our study increased the number of observations and enabled more precise estimation of associations for individual agents, including less frequently reported exposures.

Exposure data were self-reported and not externally validated; although participants were asked specifically about each agent, misclassification or underreporting was possible. Exposure timing could not be distinguished by shift, as exposures were reported at the job level and many jobs included mixed schedules. Importantly, our findings reflect the likelihood of reported exposure rather than exposure intensity or cumulative dose, and we could not infer actual exposure levels or downstream health effects from these data. While we included a wide range of potentially carcinogenic agents, some were reported by few jobs, limiting statistical power to detect associations for those exposures.

Although we adjusted for occupation and industry at the 2-digit level, residual confounding remains possible. Broad occupational codes may group together workers with substantially different responsibilities and exposure profiles. For example, within healthcare occupations, day shift jobs may disproportionately include supervisory, administrative, or senior roles with limited direct patient care or procedural responsibilities, whereas night shift roles may more frequently involve hands-on clinical or technical duties. Such role stratification within occupational categories could contribute to the observed differences in exposure likelihood (eg, ionizing radiation). Differences in workplace organization and supervision between day and night shifts may also influence workers’ awareness and reporting of potential exposures, which could contribute to the observed associations. While identifying the specific occupational or industrial subgroups driving individual exposure associations would add interpretive value, stratified analyses were not feasible given sparse observations within many subgroup-by-exposure combinations. Finally, exposure prevalence estimates reflect the historical and regulatory context of British Columbia and may not be directly generalizable to other geographic regions or more contemporary workforce structures.

In summary, night shift work was associated with a higher likelihood of exposure to multiple potentially carcinogenic agents across occupations and industries, suggesting that differential exposure to hazardous agents may contribute to the broader health disparities observed among night shift workers. Although we adjusted for occupation and industry, residual differences in job responsibilities may at least partially explain the observed associations. Future research incorporating more granular task-level information and quantitative exposure assessment will be important for clarifying the mechanisms underlying these differences.

## Supplementary material

Supplementary material

## References

[1] National Toxicology Program Cancer Hazard Assessment on Night Shift Work and Light at Night. National Toxicology Program; 2021 Apr. 34197056

[2] Haus EL, Smolensky MH. Shift work and cancer risk: potential mechanistic roles of circadian disruption, light at night, and sleep deprivation. Sleep Med Rev 2013 Aug;17(4):273–84. 10.1016/j.smrv.2012.08.003 23137527

[3] El-Zaemey S, Carey RN. Variations in exposure to carcinogens among shift workers and non-shift workers. Am J Ind Med 2019 Apr;62(4):352–6. 10.1002/ajim.22950 30680755

[4] Miguet M, Rukh G, Titova OE, Schiöth HB. Important Difference between Occupational Hazard Exposure among Shift Workers and Other Workers; Comparing Workplace before and after 1980. Int J Environ Res Public Health 2020 Oct;17(20):7495. 10.3390/ijerph17207495 33076288 PMC7602472

[5] Pepłońska B, Burdelak W, Bukowska A, Krysicka J, Konieczko K. Night shift work characteristics and occupational co-exposures in industrial plants in Łódź, Poland. Int J Occup Med Environ Health 2013 Aug;26(4):522–34. 10.2478/s13382-013-0126-y 24037502

[6] Jay SM, Gander PH, Eng A, Cheng S, Douwes J, Ellison-Loschmann L et al. New Zealanders working non-standard hours also have greater exposure to other workplace hazards. Chronobiol Int 2017;34(4):519–26. 10.1080/07420528.2017.1307850 28426386

[7] Barbey C, Weibel L, Clerc F. Coexposure of French workers to night and/or shift work and chemical substances. Ann Work Expo Health 2026 Feb;70(2):wxaf082. 10.1093/annweh/wxaf082 41348325

[8] Dhalla A, McDonald TE, Gallagher RP, Spinelli JJ, Brooks-Wilson AR, Lee TK et al. Cohort Profile: The British Columbia Generations Project (BCGP). Int J Epidemiol 2019 Apr;48(2):377–378k. 10.1093/ije/dyy160 30169793

[9] Agents Classified by the IARC Monographs. Volumes 1–141 [Internet]. [cited 2026 Apr 23]. Available from: https://monographs.iarc.who.int/agents-classified-by-the-iarc

[10] Peplonska B, Bukowska A, Sobala W. Association of Rotating Night Shift Work with BMI and Abdominal Obesity among Nurses and Midwives. PLoS One 2015 Jul;10(7):e0133761. 10.1371/journal.pone.0133761 26196859 PMC4511417

[11] Bhatti P, Mirick DK, Randolph TW, Gong J, Buchanan DT, Zhang JJ et al. Oxidative DNA damage during night shift work. Occup Environ Med 2017 Sep;74(9):680–3. 10.1136/oemed-2017-104414 28652381

[12] Zanif U, Lai AS, Parks J, Roenningen A, McLeod CB, Ayas N et al. Melatonin supplementation and oxidative DNA damage repair capacity among night shift workers: a randomised placebo-controlled trial. Occup Environ Med 2025 Mar;82(1):1–6. 10.1136/oemed-2024-109824 39993913

