# Supplementary material for: Occupational exposure to carcinogenic substances in night versus day shift jobs
Source: Scand J Work Environ Health. 2026 Jun 26;52(4):434–41. doi: 10.5271/sjweh.4307 (PMC13349335; doi:10.5271/sjweh.4307)
Supplement: Supplementary material [file SJWEH-52-434-S001.pdf]

Occupational History Questionnaire

Please tell us about **EACH job or occupation you had for at least 3 months or longer** both in Canada and elsewhere. Include full time, part time, seasonal, volunteer, and military service if you have worked the equivalent of 3 months or more. Include your current job, even if you have had it for less than 3 months or **if you are retired, we still require the information**. **Begin with your most recent job and continue back to your first job**.

1. Work History ⟶

	A	B	C	D	E	F
**Job #**	**Time Period** Start year and end year of each job	**Type of Industry, Business, or Service**	**What was your Job Title?**	**Job Location:** City, Province and Country (if outside Canada)	**Main Job Duties**	**Job Status:** -full time -part time -casual -seasonal -volunteer
Example:	*1998 to 2005*	*Automotive*	*Automotive Machinist*	*Kelowna, BC*	*transmissions and brakes, clean and repair parts*	*Full time*
**1.**	______ to______					
**2.**	______ to______					
**3.**	______ to______					
**4.**	______ to______					
**5.**	______ to______					
**6.**	______ to______					
**7.**	______ to______					
**8.**	______ to______					

Please provide your best estimate of the time period if you cannot remember exact years. **Please provide answers for all columns (A-M).**
Once you have finished with all your job entries, proceed to Section 2.

Questions continued - Please answer all questions for each corresponding job number (columns A-M)

G	H	I	J	K	L	M
**Job Shift - Most common type of shift you worked?** e.g. Regular morning starts between 3am and 6am, Regular Day start after 6am, Regular Evening ends around midnight<br>-Regular Morning -Rotating<br>-Regular Day -Split<br>-Regular Evening -Irregular<br>-Regular Night -Other	**Typical shift?**<br>-Less than 8 hrs<br>-8 hrs<br>-10 hrs<br>-12 hrs<br>-More than 12 hrs<br>-Variable<br>-Other<br>-Don't know	**#hours usually worked per week**<br>-For N/A or Don't know enter 0	**Number of weeks worked per year (0-52)**<br>-For N/A or Don't know enter 0	**How much advanced notice did your employer give you for shifts worked?**<br>-Months -Days<br>-Weeks -Hours<br>-N/A	**Was self-scheduling or shift swapping permitted by employer?**<br>-Self scheduling<br>-Shift swapping<br>-Both<br>-Neither<br>-N/A or Don't know	**How many days per month did you work Night shift?**<br>(i.e. night shift is >3 hrs between 12am-5am<br>-For N/A or Don't know enter 0
Example: Regular Day	*8 hrs*	*40 hrs*	*50 weeks*	*2 weeks*	*Shift swapping*	*0*
		______hrs/wk	______wk/yr			_______days/mo
		______hrs/wk	______wk/yr			_______days/mo
		______hrs/wk	______wk/yr			_______days/mo
		______hrs/wk	______wk/yr			_______days/mo
		______hrs/wk	______wk/yr			_______days/mo
		______hrs/wk	______wk/yr			_______days/mo
		______hrs/wk	______wk/yr			_______days/mo
		______hrs/wk	______wk/yr			_______days/mo

2. Work Exposure

Have you ever worked with or been exposed to any of the following while at a job? (Select ALL that apply)

Name of Material or Exposure	Exposed? (please check box)			Job # (enter matching Job # from 1st column on work history pages)		Total number of months exposed while at this job?	
	No	Yes	Don't know	A.	B.	A.	B.
Asbestos				Job #____	Job #____	______months	_____months
Chromium salts				Job #____	Job #____	______months	_____months
Cadmium salts				Job #____	Job #____	______months	_____months
Coal tar, soot, pitch, asphalt, creosote				Job #____	Job #____	______months	_____months
Mineral, cutting or lubricating oil				Job #____	Job #____	______months	_____months
Benzidine				Job #____	Job #____	______months	_____months
Benzene				Job #____	Job #____	______months	_____months
Isopropyl				Job #____	Job #____	______months	_____months
Dyestuffs				Job #____	Job #____	______months	_____months
Vinyl Chloride				Job #____	Job #____	______months	_____months
Degreasing				Job #____	Job #____	______months	_____months
Ionizing radiation (i.e., x-ray)				Job #____	Job #____	______months	_____months
Welding				Job #____	Job #____	______months	_____months
Diesel engine exhaust				Job #____	Job #____	______months	_____months
Gasoline engine exhaust				Job #____	Job #____	______months	_____months
Pressure treated wood				Job #____	Job #____	______months	_____months
Paints, stains, varnish				Job #____	Job #____	______months	_____months
Glue				Job #____	Job #____	______months	_____months
Herbicides, insecticides, fungicides				Job #____	Job #____	______months	_____months
Other (not listed above) Specify________________________				Job #____	Job #____	______months	_____months
